# Reprocessed magnetorheological elastomers with reduced carbon footprint and their piezoresistive properties

**DOI:** 10.1038/s41598-022-16129-y

**Published:** 2022-07-14

**Authors:** A. Munteanu, A. Ronzova, E. Kutalkova, P. Drohsler, R. Moucka, M. Kracalik, O. Bilek, S. A. Mazlan, M. Sedlacik

**Affiliations:** 1 Centre of Polymer Systems, University Institute, Tomas Bata University in Zlín, Trida T. Bati 5678, 760 01 Zlín, Czech Republic; 2 Department of Production Engineering, Faculty of Technology, Tomas Bata University in Zlín, Vavreckova 275, 760 01 Zlín, Czech Republic; 3 Polymer Centre, Faculty of Technology, Tomas Bata University in Zlín, Vavreckova 275, 760 01 Zlín, Czech Republic; 4 Institute of Polymer Science, Johannes Kepler University Linz, Altenberger Straße 69, 4040 Linz, Austria; 5 Engineering Materials and Structures (eMast) iKohza, Malaysia-Japan International Institute of Technology (MJIIT), Universiti Teknologi Malaysia, Jalan Sultan Yahya Petra, 54100 Kuala Lumpur, Malaysia

**Keywords:** Engineering, Materials science

## Abstract

Despite the vast amount of studies based on magnetorheological elastomers (MREs), a very limited number of investigations have been initiated on their reprocessing. This paper presents a new type of recyclable MRE which is composed of thermoplastic polyurethane (TPU) and carbonyl iron particles (CI). The chosen TPU can be processed using injection moulding (IM), followed by several reprocessing cycles while preserving its properties. Numerous types of injection moulded and reprocessed MREs have been prepared for various particle concentrations. The effect of thermo-mechanical degradation on the recycled MREs has been investigated while simulating the reprocessing procedure. An apparent decrease in molecular weight was observed for all the examined matrices during the reprocessing cycles. These changes are attributed to the intermolecular bonding between the hydroxyl groups on the surface of the CI particles and the matrix which is studied in depth. The effect of reprocessing and the presence of magnetic particles is evaluated via tensile test, magnetorheology and piezoresistivity. These characterization techniques prove that the properties of our MREs are preserved at an acceptable level despite using 100% of recyclates while in real applications only up to 30% of recycled material is generally used.

## Introduction

Smart or intelligent materials are able to substantially change their material properties under an external stimulus, such as stress, electromagnetic radiation, pH, electrical or magnetic field^1,2^. A unique type of such smart materials are the magnetorheological elastomers (MREs) which are consisted of magnetic microparticles embedded in an elastomeric matrix. These composites are mostly known for their controllable viscoelastic character which can be tuned with an external magnetic field^3^. As a result, these elastomers can be used in a huge variety of applications. The most common field for applications is engineering which involves vibration absorbers, actuators and dampers^4,5^. Other notable applications include electromagnetic shielding, sensors and flexible electronics^6–8^. Lately medical applications use the MREs as fluid transportation, artificial muscles and cell substrates^9–14^. It is clear that the usage of the MREs constantly increases which arises the need of reusability of these elastomers. Recycling is an important aspect of product design from both environmental and economical point of view. However, not many MREs are investigated in this regard, as most of them are not able to undergo such procedures.

A good solution for these elastomers is the selection of an appropriate filler and matrix with the ability to undergo several recycling processes. Carbonyl iron (CI) is the most common filler used in MREs due to its superior magnetic properties and stability^15^. In terms of reprocessing, exposure to high temperatures can lead to inferior magnetic particles, however polymers are usually reprocessed at much lower temperatures^16^. A sufficiently flexible elastomer including synthetic silicone matrices^17^, nitrile rubbers^18^, and polyurethanes^19,20^ represent the second important component of the most common MREs. The complexity of reusing these MREs is related to the crosslinked structure of the composite’s matrix. In contrast to crosslinked matrices, thermoplastic elastomers (TPEs) can be melted and reused^21,22^. Thermoplastic elastomers can compete with vulcanized rubbers at room temperature in terms of their mechanical properties and difficulty of processing. The main difference between TPE and vulcanized rubbers can be found in the structure of the polymer network. Vulcanized rubbers share stronger networks, a property obtained from the disulfide bonds, however they are not fit for reprocessing. The balanced properties of the TPEs originate from their microstructure, which is generated by alternating mutually immiscible soft and hard elastomeric segments with a distinctly different glass transition temperature^23^.

Considering the broad utilization of MREs, they have to be highly functional and tide over some difficulties. The properties of the MREs are greatly affected by the magnetic properties of the particles, their concentration and microstructure which is either a well-dispersed isotropic state or directed in an anisotropic arrangement^24^. In addition, the flexibility of the matrix is crucial for maintaining specific properties for example the MR effect^7^. The MR effect describes the difference of the matrix’s stiffness before and after the application of a magnetic field^25^. Most studies use the shear storage (*G'*) and the loss modulus (*G″*) to evaluate the MR effect^26,27^. An excess amount of particles in the matrix enhances the MR effect however, highly filled MREs usually suffer from the Payne effect which could significantly limit their applications. The Payne effect is observed as a simultaneous rapid decrease of the *G'* and a local maximum of *G″* above certain values of deformation. It is a common behaviour for elastomers embedded with a filler at high concentrations and it is based on changes in the microstructure of the material^28^. Nevertheless, this phenomenon is rather ignored in the literature dealing with MREs and thus needs to be further investigated.

In this study, we use a thermoplastic polyurethane (TPU) as a MRE which can be processed and recycled while competing with its analogous in terms of MR performance. These properties of the TPU originate from its structure which is composed of a two-phase chemically bonded soft and hard segments^29^. TPU-based composites are used in a wide range of applications^30–32^ however, their presence in MREs is rather limited^20,33,34^. We simulate the processing conditions and evaluate their effects on the structure and molecular properties of the matrices. In addition, we characterize the samples in terms of mechanical and MR performance using industrial-friendly equipment. Lastly, we prove that these MREs are suitable for piezoresistive applications. Piezoresistive sensors have been investigated for decades^35,36^ leading to a significant improvement in a variety of state-of-the-art robotics^37^. Our MREs are able to detect external forces under deformation converting them into a change of resistivity thus allowing them to be used as sensors.

## Experimental

### Materials and fabrication of the MREs

The CI particles (CN grade, iron content > 99.5%, *d*_50_ = 6.5–8.0 μm; BASF, Germany) were used as the magnetic filler for the preparation of MREs. Elastolan® 35A12P (BASF, Germany; 37 Shore A hardness) was chosen as an appropriate TPU matrix. The MREs were prepared by mixing the CI particles with the TPU matrix in various concentrations from 30 to 80 wt%. The samples with the most diverse and representative results are presented here including 30, 50 and 80 wt%. The mixing was performed using a twin-screw counter-rotating mixer supplied by Brabender (Duisburg, Germany). Each matrix was prepared at 170 °C, following 1-min dosing of the mixture and its compounding for 4 min at 50 rpm. In addition to composite samples, a neat matrix was also subjected to the mixing process in order to investigate the effect of its degradation and to compare its mechanical properties with the MREs. After cooling, the homogeneous matrices were cut into small pieces so they can be processed with IM. The IM was executed using HAAKE MiniJet Pro—Piston IM System (Thermo Scientific, Germany) and disk-shaped samples of 25 and 1.23 mm in diameter and height, respectively, and standard dog-bone tensile specimens (type 5 according to ISO 527) with the same thickness were produced. During the IM of filled samples, the optimal parameters, such as the temperature of the cylinder, temperature of the mould, the injection pressure/time and post-pressure/time, were set up 190 °C, 30 °C, 450 bar/7 s, and 350 bar/3 s, respectively. The same parameters were set up for neat samples (TPU) with the exception of the temperature of cylinder which was set at 180 °C. The prepared samples were labelled as raw material (R0) and characterized by the methods below. Consequently, the samples were further cut into small pieces, compounded, injected under the same conditions as stated above, and labelled as recycled material (R1). The whole process of mixing and IM was repeated 3 times thus fabricating recycled samples labelled R1–R3.

### Degradation process analysis

The effect of recycling on the pure TPU matrix was studied by Fourier-transform infra-red spectroscopy (FTIR). The measurements were performed on FTIR instrument Nicolet 6700 (Nicolet, USA) supplied with an ATR-accessory with a diamond crystal under laboratory temperature with the spectra resolution of 2 cm^–1^ from 64 scans, in the range of 4000–700 cm^–1^, however the region between 2000 and 2800 cm^–1^ is not presented due to the intrinsic absorption of the diamond crystal. The weight average molecular weight (*M*_w_), the number average molecular weight (*M*_n_), and the polydispersity index (*Ð* = *M*_w_*/M*_n_) of the tested samples were determined from the peaks corresponding to the polymer fraction according to the absolute calibration method by the gel permeation chromatography (GPC) method using a Waters HPLC system, equipped with a Waters model e2695 and a Waters model 2414 differential refractometer (Waters Corporation, Milford, USA). The samples were dissolved in tetrahydrofurane (THF) (2–3 mg mL^−1^), then stabilized with butylated hydroxytoluen (BHT) (240 mg L^–1^) and lastly, filtered using a syringe filter (0.45 μm). The separation was carried out using a series of gel-mixed bed columns (Polymer Laboratories Ltd, Shropshire, UK) as follows: 1 × PLgel-Mixed-A bed column (300 × 7.5 mm, 20 μm), 1 × PLgel-Mixed-B bed column (300 × 7.5 mm, 10 μm), and 1 × PLgel-Mixed-D bed column (300 × 7.5 mm, 5 μm); the mobile phase containing the THF was stabilized with BHT (240 mg L^–1^) at 40 °C. The flow rate of the mobile phase was set to 1.0 mL min^−1^ and the injection volume equal to 100 μL. All data were analysed using the Empower 3 software.

The reflection spectra of the TPU samples with the same dimensions as used in rheological analysis (see below) were analysed using a Lovibond RT850i (Tintometer Ltd) equipped with xenon pulse light. For the colorimetric evaluation the matrices were taken from the original pellet and then processed through IM. The measurement of reflection spectra in the range of 360–750 nm was performed with a 10‐mm aperture size and d/8° observation geometry. The average whiteness index (WI) was evaluated from the obtained spectrum by averaging 10 measurements at various positions on the sample according to ASTM E313-20. The glass transition temperature (*T*g) of the soft segments of the matrices was investigated via a differential scanning calorimetry (DSC) method using the Gas Controller GC100 (Mettler Toledo, Switzerland). The samples were prepared with similar weights (≈ 6 mg) and measured for a temperature range of – 40 to 250 °C using heating and cooling rates of 20 °C min^–1^ under nitrogen atmosphere. The *T*g of the soft segments was evaluated using the half-height technique in the transition regime. The error for each *T*g was taken as the average temperature step for each measuring point.

### Rheology

The rheological properties of the TPU elastomers were characterized using the rotational rheometer Physica MCR 502 (Anton Paar, Graz, Austria). The magnetorheological measurements were performed using a magneto-cell (Physica MRD 180/1 T). The samples were exposed to various external magnetic fields up to 750 kA m^–1^. To perform the degradation study, a water-cooled Peltier system (Physica H-PTD 200) was equipped, which enables one to reach temperatures up to 180 °C to simulate the processing conditions. For both accessories, a parallel-plate geometry was used. The diameters of plates were 20 and 25 mm for the magneto-cell and the Peltier, respectively. In addition, the geometry used for magnetorheological measurement was sandblasted and 0.5 N normal force was applied during the measurement for the elastic samples to eliminate any possible wall-slip at low shear rates. All measurements were performed in the linear viscoelastic regime (LVE) where the modulus is independent of strain. The LVE was identified using dynamic strain sweeps for strains between 0.001 and 10% at the frequency of 1 rad s^–1^. At elevated temperatures, the samples were tested under inert atmosphere using nitrogen. Finally, dynamic time sweeps were performed at 1 rad s^–1^ until the elastomers reached a steady state for samples unaffected by thermal degradation. For the neat samples, further frequency sweeps were performed in the 0.1–100 rads s^–1^ regime.

### Mechanical testing

The samples for tensile tests were produced directly in the shape of the test specimen according to the requirements of the standard test method using the IM process. The tensile properties of the TPU matrix as well as the MREs were investigated using the tensile testing machine M350-5 CT (Testometric Company, Lancashire, UK) with a cross-head speed of 500 mm min^–1^. The measurements were performed according to the ASTM D638 standard test method at the room temperature and subsequently the results of the tensile strength were evaluated as an arithmetic mean using 5 samples of type 5. The standard deviation was obtained using five test specimens from the stress–strain dependencies. The coefficient of variation among all the tested variants of samples was lower than 10% and 12% for tensile strength and for Young's modulus, respectively.

### Piezoresistivity testing

To determine the piezoresistive properties, an electrometer (Keithley 6517A, USA) coupled with a tensile machine (M3750-5CT, Testometric, UK) and supplied with a load cell with a maximum capacity of 5 kN was used. Cylindrical samples with 4 mm high and 10 mm in diameter were sandwiched between two gilded brass electrodes and were gradually compressed narrowing their mutual distance by up to 25% of their initial spacing (sample height) at the rate of 5 mm min^–1^. The deformations were set to 5, 10, 15, 25% of the height of the sample and were kept for the period of 1 min during which conductivity (later converted to resistivity as a reciprocal value) was measured. The measurements were performed at room temperature. The conductivity (σ) was calculated from the measured current–voltage dependencies according to the following formula:

1 $$\sigma =\frac{I}{U}\frac{t}{A}$$

where *t* is the sample thickness (distance of electrodes) for given deformation, *A* is the nominal area of the sample (electrode), *I* is the electric current, and *U* is the voltage.

## Results and discussion

### Degradation process analysis

**Figure 1:**
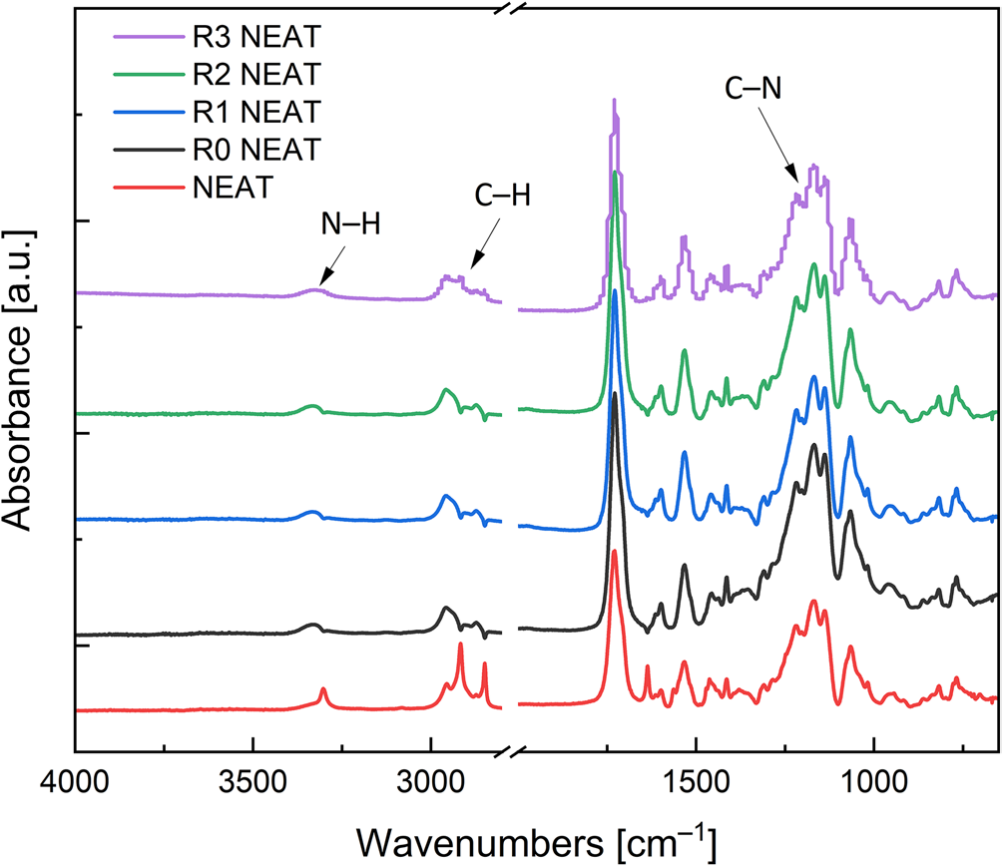
FTIR analysis of neat TPU matrix for each reprocessing cycle.

The degradation of TPU was examined by FTIR spectra for each reprocess cycle. In Fig. 1, the reprocessing cycles spectra of neat TPU matrix can be observed. The graph displays a peak at 3300 cm^–1^ which represents an N–H stretching. It is clear, that the N–H group is reduced during reprocessing. Moreover, the figure shows characteristic peaks at 2900 and 2800 cm^–1^ of asymmetric and symmetric C–H stretching, respectively. These peaks drop with each reprocess cycle which can be attributed to the mechanical and thermal degradation.

In addition, Fig. 1 displays a peak at 1700 cm^–1^, which corresponds to an ester linkage, which is typical during the reprocessing of such matrices^38^. Finally, aromatic amine C–N groups were identified from the peaks between 1300 and 1250 cm^–1^ which are more pronounced with each recycling. The reason behind the C–N stretching is explained in detail later on. It can be concluded that FTIR spectra prove certain chemical changes in the matrix during reprocessing.

#### Gel permeation chromatography

Gel permeation chromatography analysis is an important method for the determination of the polymer's molecular weight and molecular weight distribution, which can be used to track the degradation process during processing. Table 1 shows the changes in the chain length, expressed as *M*_w_ for different recycling cycles. The effect of magnetic filler on chemical changes during IM process, followed by three processes of recycling is observed using the GPC method. In general, heat treatment of neat TPU induced a reduction in *M*_w_ caused by thermal degradation of the polymer chains. However, during the 2nd and 3rd recycling process, these values became almost unchanged, probably reaching equilibrium originating from the simultaneous degradation and accumulation of processes in the TPU^23,39^.

**Table 1:** Molecular weights and polydispersity index obtained from GPC measurements.

	Treatment	*M*_n_ (g mol^–1^)	*M*_w_ (g mol^–1^)	*Ð* (–)
R0 NEAT	IM	63,600 ± 2100	131,600 ± 2100	2.07 ± 0.06
R1 NEAT	IM/one times recycling process	58,600 ± 900	119,900 ± 700	2.05 ± 0.05
R2 NEAT	IM/two times recycling process	57,300 ± 700	115,600 ± 600	2.02 ± 0.01
R3 NEAT	IM/three times recycling process	57,700 ± 1200	115,200 ± 1300	2.00 ± 0.02
R0 80 wt%	IM	53,100 ± 4200	98,800 ± 2200	1.87 ± 0.11
R1 80 wt%	IM/one times recycling process 80 wt% filled	55,600 ± 900	100,600 ± 200	1.81 ± 0.03
R2 80 wt%	IM/two times recycling process 80 wt% filled	50,800 ± 300	88,600 ± 100	1.74 ± 0.01
R3 80 wt%	IM/three times recycling process 80 wt% filled	46,400 ± 900	81,500 ± 1100	1.76 ± 0.01

An apparent decrease in *M*_w_ and *Ð*, was observed for the 80 wt% composite in contrast to the TPU sample, indicating a chain shortening due to the presence of CI particles. In the first recycling cycle, higher values of *M*_w_ and *M*_n_ were observed. This phenomenon indicates the probable bonding of the chains with the particles during the thermal stress in the composite processing. As the number of melt agitations increases, the chains shorten and the *M*_w_ and *M*_n_ values decrease, indicating that the degradation processes prevail over the recombination^40^. The abovementioned results revealed that excessive mixing of the melt with CI particles resulted in a more significant reduction of the molecular weight for the 80 wt% polymer by up to ~ 18%, rather than the neat TPU where the reduction was only ~ 13%.

#### Colorimetry evaluation

**Figure 2:**
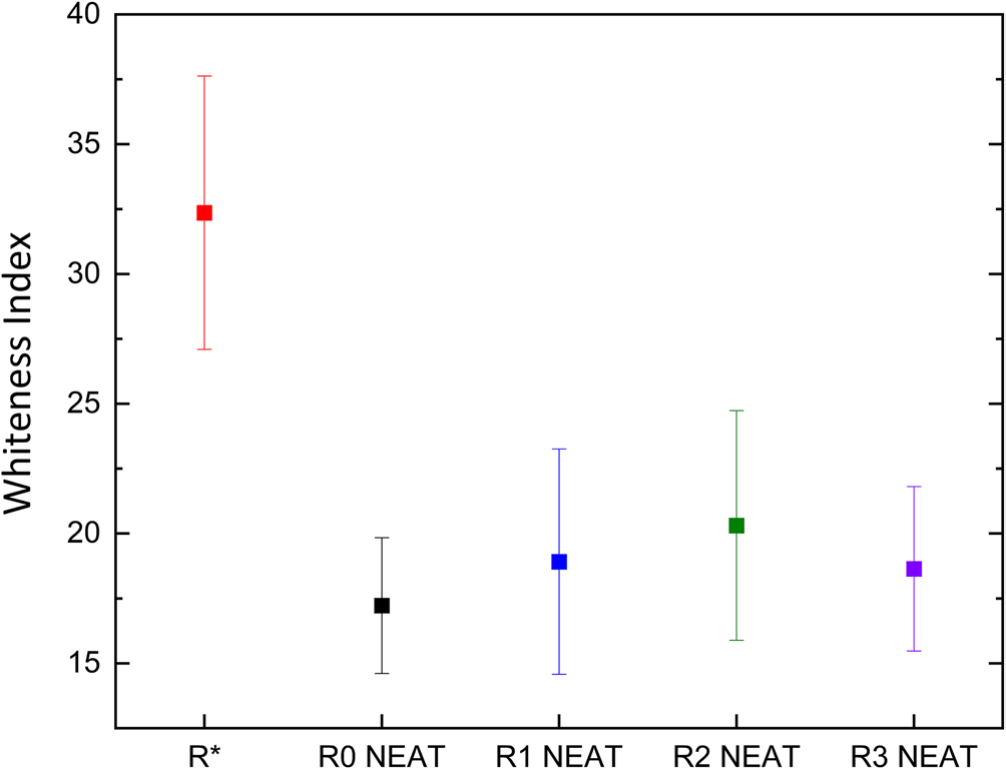
Whiteness Index values for the original pellet (R*), matrices obtained through IM process (R0) and further reprocessing (R1–R3).

An additional degradation study, relevant to the industry, was conducted through whiteness index (WI) measurements. In Fig. 2, the values of the WI are demonstrated for the same matrix during different processing states. Initially, the WI dropped by half after the matrix underwent the first IM process. This could be attributed to the degradation of the elastomer and the by-products of processing as mentioned above. Thereafter, the recycled samples share very similar values of WI indicating the same overall product. The filled matrices could not be measured due to the high concentrations of the particles. To conclude, despite this method being mainly complementary, the colour assignment for recycled products is a quick and a practical test.

### Mechanical testing

**Figure 3:**
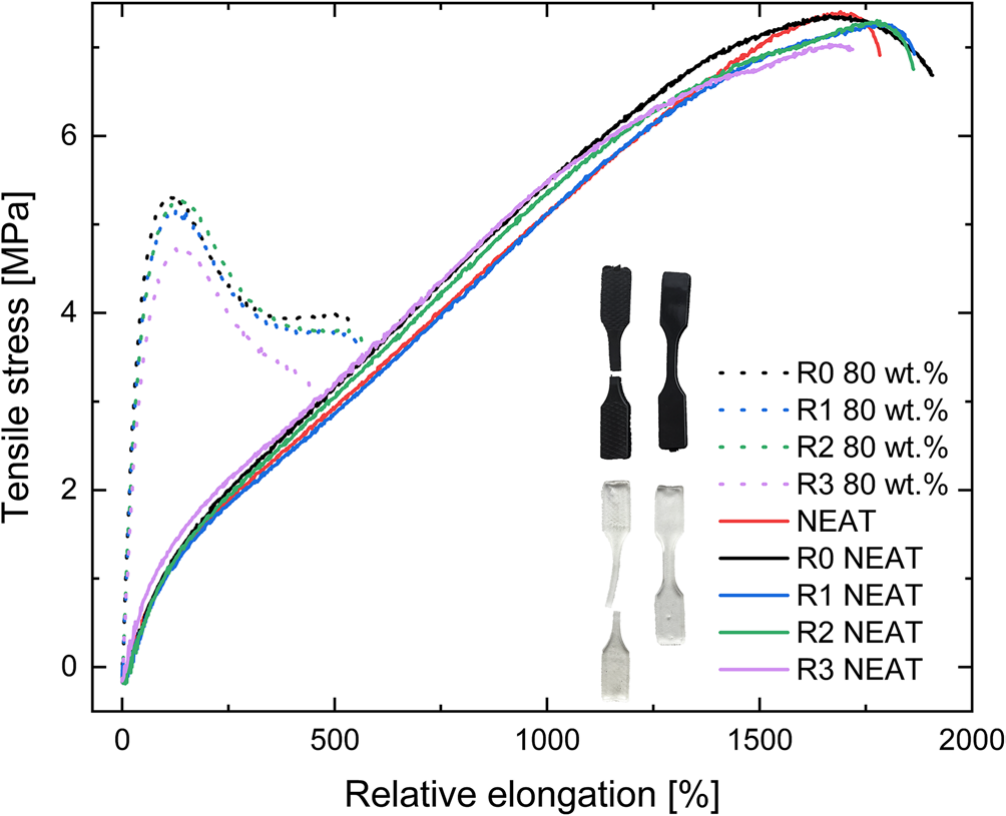
Tensile strength properties for unfilled neat TPU matrix (full line) and 80 wt% filled TPU matrix (dot line).

Tensile strength tests were conducted to study in depth the mechanical behaviour of the neat and filled TPUs. The tensile properties were measured five times for each processing cycle. The most representative stress–strain curves for each sample for both neat and filled matrices are displayed in Fig. 3. The trend of the curves for neat matrix demonstrates that the samples break at high elongations. This indicates that the matrix is consisted mostly of elastomeric segments as these curves are typical for a rubber-like materials. The MREs on the other hand, break at shorter elongations as the magnetic particles cannot deform above a certain point forcing the matrix to be extended, thus the deformation that the polymeric part feels is greater than the one that the machine imposes. All the MREs samples certainly show a very similar strain hardening and necking which ends up in a break at similar values of stress.

The maximum elongation at break differs for every recycling process of the neat TPUs, however the differences between the samples is negligible, only R3 possesses a little bit lower elongation. It can be clearly concluded from the Fig. 3 that the selected matrix is suitable for reprocessing as the matrices retain their mechanical properties during each reprocessing cycle. As shown in the graph, the filled matrix has a very similar trend to the R0 after the first two reprocessing cycles with virtually the same maximum elongation and slightly lower tensile stress.

On the other hand, the third recycling cycle shows significantly lower both elongation at break and tensile stress, which can be attributed to the degradation process caused by recycling and potential bonding between the matrix and the CI particles during the reprocessing.

**Figure 4:**
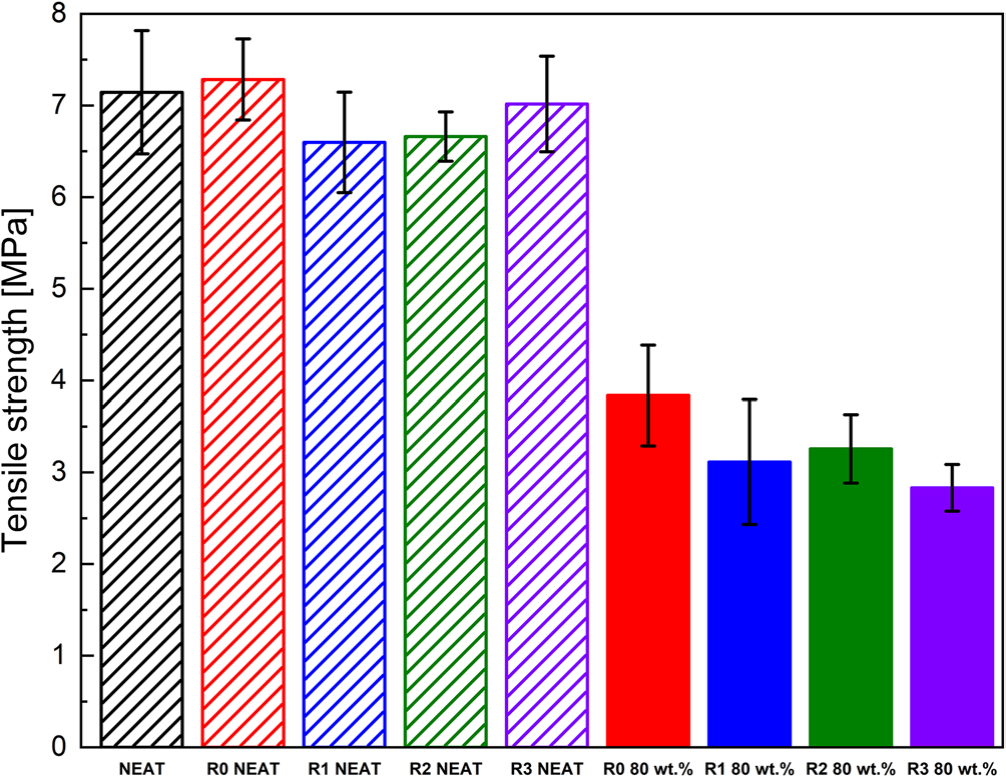
Tensile strength of the neat and 80 wt% filled TPU samples.

**Figure 5:**
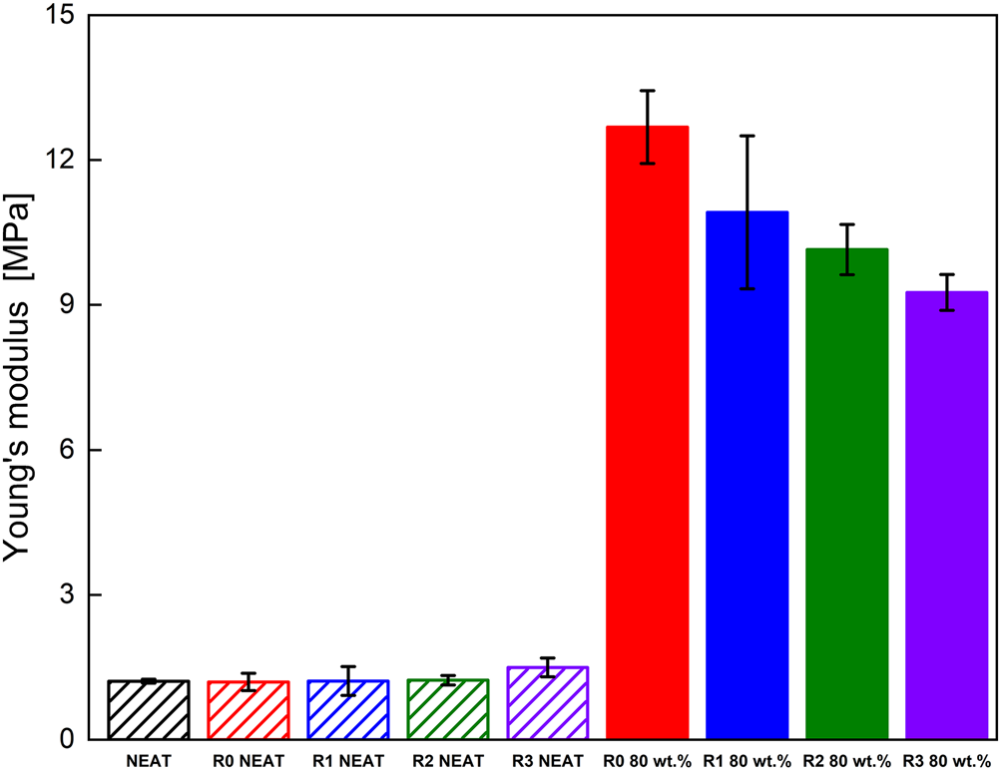
Young's modulus of the neat and 80 wt% filled TPU matrices.

Additionally, the comparison of the neat and filled TPUs reveals qualitatively different behaviour. The first difference can be seen at post-yield behaviour where the unfilled matrices have no visible strain softening whereas the filled material has distinctive stress drop beyond yield point. Furthermore, as can be observed from the Fig. 3, the unfilled materials are capable of sustaining enormous elongation with partial reversible deformation, unlike the filled analogues, in which the presence of the CI particles resulted in three times lower elongation at break. This disparity is caused by the high filling of the matrix, and the presence of the CI particles which also causes the higher fragility of the filled system. The tensile strength was also greatly reduced as shown in Fig. 4, however, the ultimate strength was less affected. Furthermore, Fig. 5 confirms that the filled matrices need higher stress to achieve the same deformation resulting in a higher Young's modulus for the filled elastomers.

Analysing the Young's modulus of the filled and unfilled samples, it can be concluded that the neat matrices have almost the same Young's modulus for all recycling cycles despite the small degradation. On the contrary, for the filled samples, a light drop of Young's modulus is observed with each recycling. It is generally known, that rigid particles increase Young's moduli of elastomers^41^, in this case it can be also assumed that with an application of external magnetic field the moduli would increase even higher^42^ which contributes to their future applications and will be shown later on.

### Rheology and glass transition temperature

**Figure 6:**
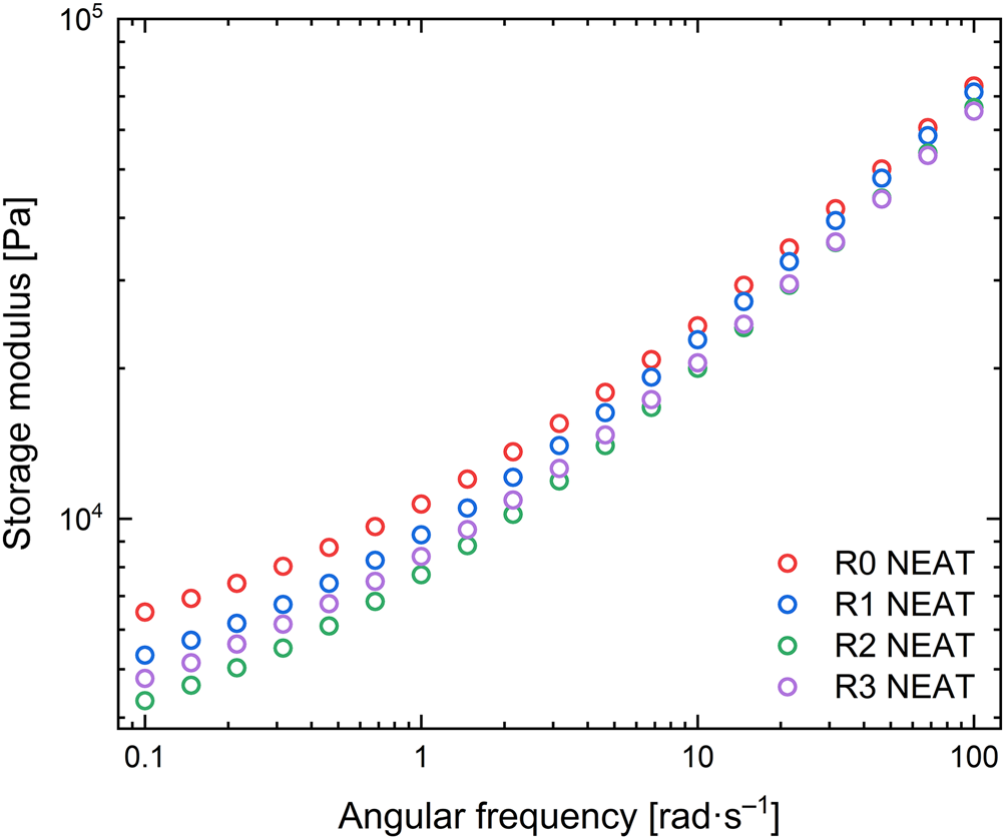
Dynamic frequency sweeps for the neat TPU samples at 150 °C.

To compare the structure of the recycled neat TPU matrices, dynamic frequency sweeps were performed at 150 °C, as shown in Fig. 6. At high frequencies, the recycled matrices show no difference from each other. It seems that the values of *G'* for these samples will converge at even higher frequencies. On the other hand, the recycled samples show a reduced *G'* at lower frequencies, however the difference is not very significant. This decrease is not proportional to the reprocessing cycles and the elastic modulus differs approximately by 10% with each cycle. Generally, the *G'* is reduced except for the case of R2. This behaviour is known to generally follow the tensile stress trend^40^. The abovementioned trend is the same as the tensile stress in Fig. 6. A possible explanation of the drop could be associated with the degradation of the TPU matrix as mentioned above^29^. The shorter chains would disentangle faster for matrices with lower molar mass. Another possible explanation would be the oxidation of the outer layer of the matrices which could decrease the material’s strength during processing^43^. Nevertheless, the outcome of the changes caused by the third recycling process in terms of *G'* seems to be minor from a practical point of view.

**Figure 7:**
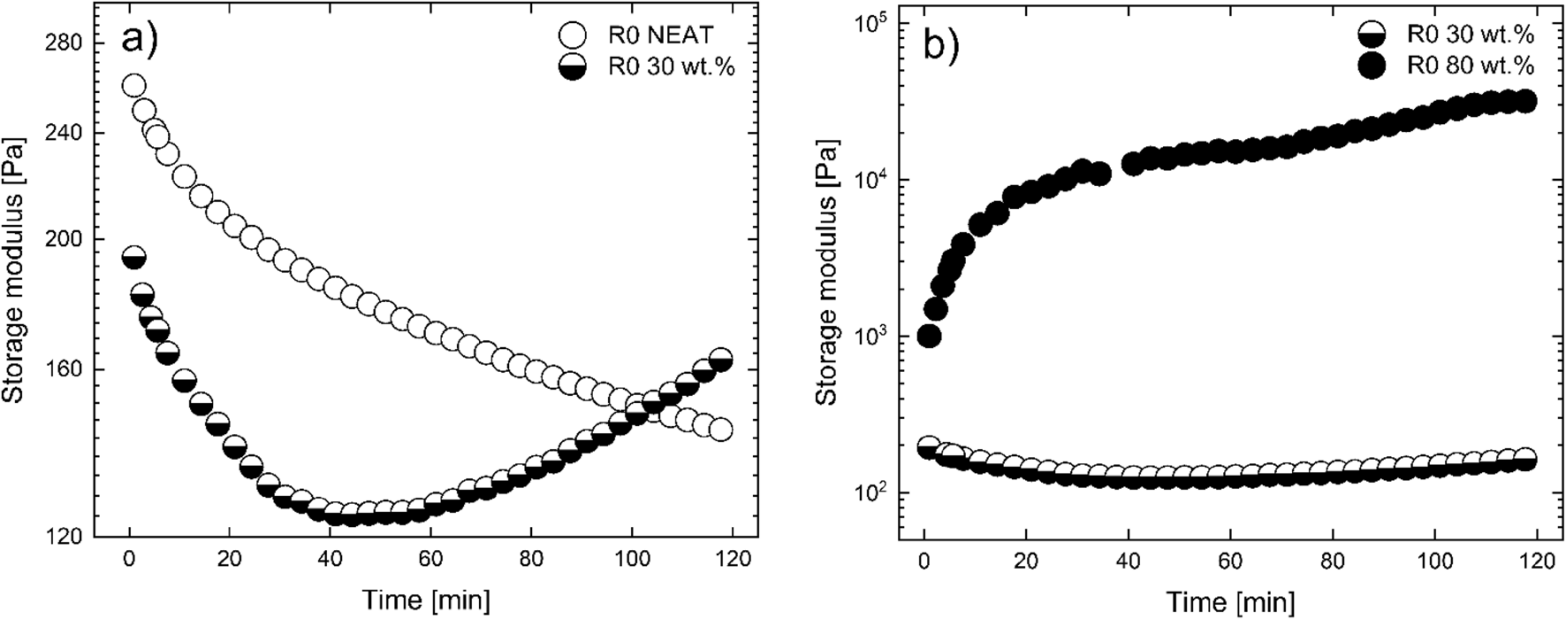
Dynamic time sweeps for (**a**) R0 PURE open circles and R0 30 wt% half open circles and (**b**) R0 30 wt.% half open circles and R0 80 wt.% filled circles.

In order to simulate the sample during the recycling process, dynamic time sweep tests (angular frequency 1 rad s^–1^ and strain within LVE) were performed at 170 °C for specific samples. In Fig. 7, the neat TPU matrix is compared with their filled analogue. The neat matrix shows a typical polymeric behaviour with the *G'* decreasing with time until the chains are relaxed and *G'* becomes independent of time. The abovementioned behaviour could hinder a degradation process; however, it cannot be evaluated for this matrix with this method. Moreover, based on the last observation, it can be confirmed that a potential curing or crosslinking process (if any) is insignificant due to the decreasing trend of *G'*. The 30 wt% filled matrix, on the other hand, shows a similar drop of *G'* for the first 50 min before a noteworthy increase of *G'* is observed. This can be attributed to the covalent bonding which occurs between the bare CI particles and the matrix^44^. These particles are covered with hydroxyl groups at their surface which can react with the end-groups of the TPU chains^45,46^. The abovementioned statement is further supported by *Fig. **7** b)* in which two MREs with different concentrations are compared. It can be clearly seen that for the 80 wt% filled matrix, the increase of the *G'* starts immediately and is orders of magnitude higher than the 30 wt% MRE. In addition, the behaviour for the 80 wt% is very important in the present study as this increase takes place during the same time-window as the fabrications and recycling process. On the other hand, for 30 wt% this increase can be considered insignificant as it can only be noticed after 50 min, which corresponds to more than ten reprocessing cycles.

It is worth mentioning that a similar test was performed with the sample exposed to O_2_ by removing the hood which provided a N_2_ based environment. A sharp increment of *G'* was observed for all samples due to reactions with oxygen. The latter behaviour can lead to inferior magnetic particles formation (iron oxidation), but such by-products are formed at much higher temperatures^16^. In Fig. 7 though, all matrices are under dormant conditions.

**Figure 8:**
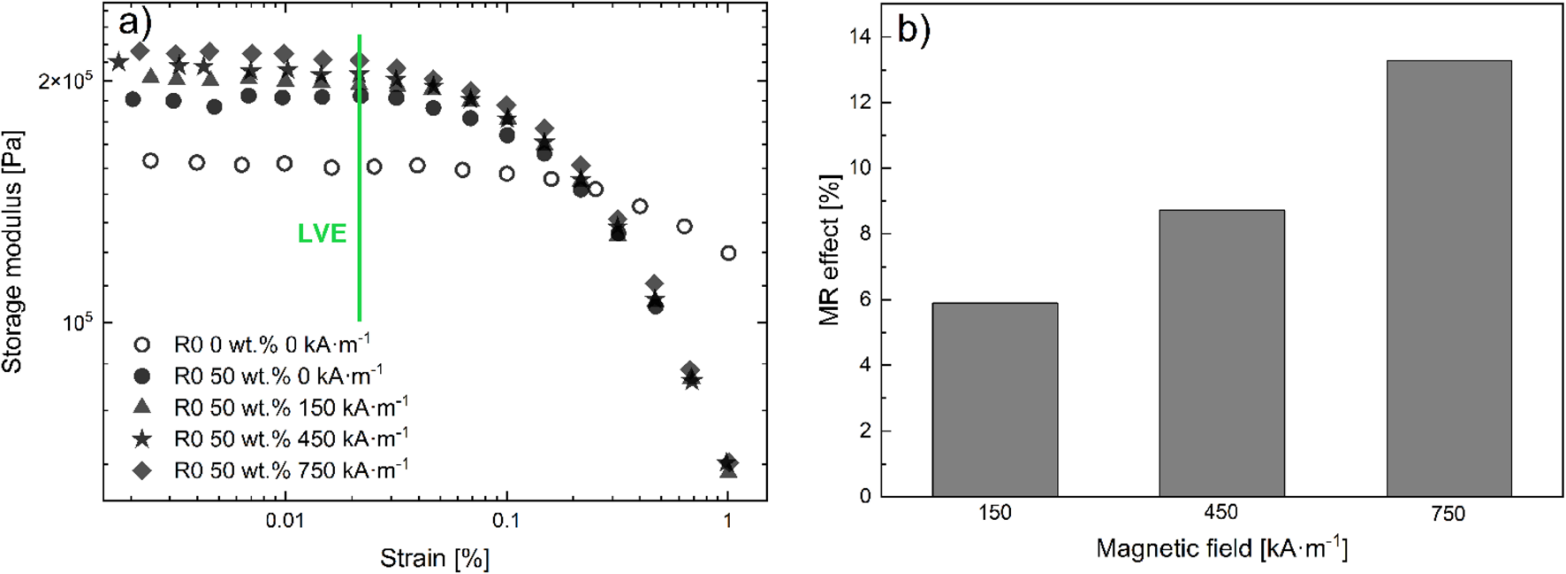
(**a**) Dynamic strain sweeps for samples containing 50 wt% CI particles at different magnetic fields (filled symbols; circles 0 kA m^–1^; triangles 150 kA m^–1^; stars 450 kA m^–1^; diamonds 750 kA m^–1^); neat TPU matrix (unfilled) and (**b**) MR effect for the same 50 wt% matrix.

The *T*g of the soft segments was evaluated using the DSC method as it is a critical parameter for magnetorheology^34^. The *T*g values for the TPU 80 wt% MREs are shown in Table 2. An apparent increase of *T*g is observed after the first reprocessing. There are two main reasons for the *T*g to increase. The first indicates an incensement in *Mw*, however, considering the GPC measurements that is not the case. The second and most probable reason is the limited mobility of the chains or their stiffening. It has been reported that TPU matrices are prone to intermolecular bonding during processing^47^. It has been observed for particles other than CI to bond with this matrix^48^. As a result, the mobility of the chains is reduced leading to higher values of *T*g. As shown in Fig. 8b, the possible bonding seems to be intense only during the first minutes of processing. This is attributed to the rate that *G'* increases which is constantly dropping and is indicated by the concave curvature. This is in agreement with the *T*g values reaching an equilibrium after the second reprocessing which corresponds to at least 10 min of processing not counting the time to cool down. Lastly, the values of the *T*g have a significant influence on the MR effect. For a different TPU with a similar *T*g but filled with the same concentration of CI particles, a 9 °C increase of the *T*g corresponded to a ~ 70% decrease of the MR effect^34^. For the recycled samples, the bonding between the particles and the matrix suppresses the MR performance, thus only injection moulded matrices were studied. To conclude, the abovementioned bonding reduces the effects of degradation on the mechanical properties, and the MR effect is suppressed for the recycled matrices. However, in the real applications, only one third of the matrix will be recycled thus the MR performance of the injection moulded samples will not be changed dramatically.

**Table 2:** The Tg values of the soft segments at different reprocessing cycles for the 80 wt% MRE.

	R0	R1	R2	R3
*Tg* (± 0.3 °C)	– 17.4	– 13.1	– 14.5	– 14.4

The magnetic properties of the TPU matrices filled with CI particles were evaluated at room temperature using dynamic strain sweeps under various homogeneous magnetic fields through the MR effect which is defined in the following formula:

2 $${MR}_{\mathrm{effect}}=\frac{{G\mathrm{^{\prime}}}_{\mathrm{on}}-{G\mathrm{^{\prime}}}_{\mathrm{off}}}{{G\mathrm{^{\prime}}}_{\mathrm{off}}}$$

where the on- and off-states are referred to as the presence and absence of the magnetic field, respectively. Generally, samples with particle concentrations below 50 wt% do not show any significant MR effect which was observed in the past for a very similar system^33^. On the other hand, the 80 wt% matrices are too immobile due to the bonds with the matrix and therefore an insignificant MR effect was observed. For that reason, a new matrix filled with 50 wt% was prepared and the MR performance was studied through dynamic strain sweeps. Figure 8a shows an increase in *G'* when the samples are exposed to external magnetic fields. The increase of *G'* is attributed to the magnetic particles attempting to align themselves and form chain-like structures parallel to the magnetic field. In addition, this trend is present mainly during the LVE where *G'* is independent of strain, which can be distinguished by the green solid line in Fig. 8a. Above the critical strain, the current structure collapses indicated by the sudden drop of the *G'*. The data in the LVE seems scattered especially at lower strains which is attributed to the high filling and stiffness of the matrix. Another difficulty is connected with the values of torque at lower strains which are only a few times higher than the resolution of the instrument. The latter is commonly encountered for highly filled matrices^49^. As a result of the slightly scattered data, the average of these points was considered for the evaluation of the MR effect. Unlike the responsive LVE, the magnetic field does not affect the modulus soon after the critical strain is reached. Thus, the MR effect for various fields was obtained only from the LVE. A linear incensement of the MR effect is demonstrated in Fig. 8b. These results can be compared with similar elastomers, thus these processed TPU are able to compete and surpass other TPU-based MREs on the MR effect point of view^33,34^.

Lastly, it is very important to address the Payne effect as it could become a potential problem for future applications. Despite being observed in MREs and highly filled matrices as a sudden decrease in *G'*, similar to the one in Fig. 8a, this effect is completely ignored in most of the studies regarding MREs. For the MREs specifically, it experiences more effects such as a steady increment of *G'* with each consequential measurement making it worth investigating^28^. However, the focus of this study is to only evaluate the presence of the Payne effect. In Fig. 8a, the neat TPU matrix is also illustrated with a similar overall behaviour as the filled samples. The two main differences include the values of *G'* in the LVE which are higher for the filled particles and the extended LVE regime of the neat matrix. Both observations are common for highly filled matrices^50^. Since the pure matrix also shows a drop in the neighbourhood of the filled particles it is safe to conclude that any potential Payne effect could exist only in the limited strain regime around 0.02 and 0.2% where the LVE is terminated for the filled and neat matrices, respectively. However, as mentioned before, matrices with high filler concentrations have their LVE shorten^50^. As a result, the drop in *G'* is mainly caused by the collapse of the polymeric structure, rather than the collapse of the filler’s network which should be more robust regardless due to the particle–matrix bonding of the matrix as mentioned above.

### Piezoresistivity testing

**Figure 9:**
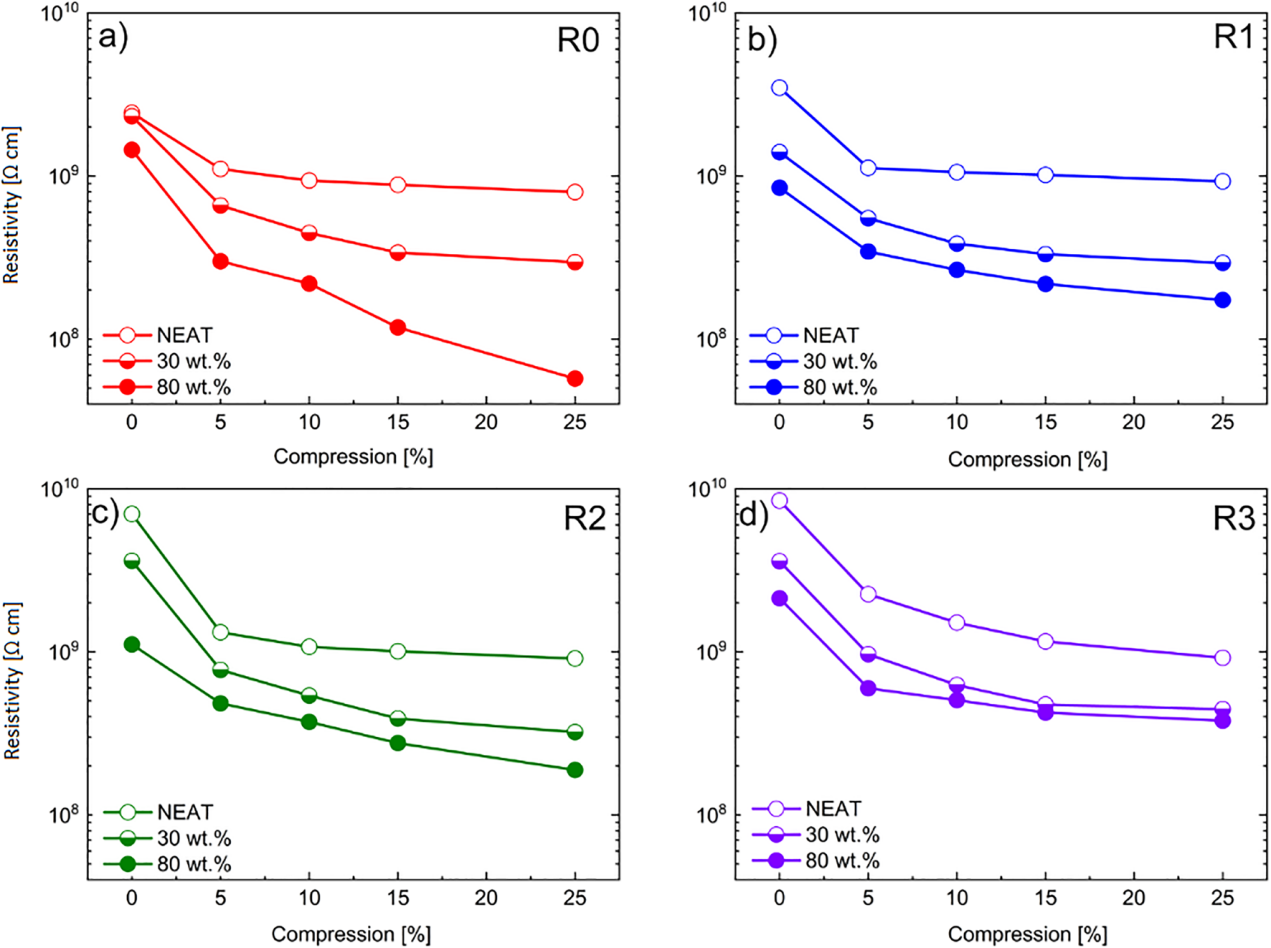
Resistivity dependences on the relative compression deformation of the neat TPUs (open symbols), 30 wt% filled TPUs (half up open symbols) and 80 wt% filled TPUs (solid symbols) for (**a**) IM/samples (red), (**b**) IM/one time recycling process (blue), (**c**) IM/two times recycling process (green), (**d**) IM/three times recycling process (purple).

In general, when a conductive composite is exposed to compression, its conductivity is increased (resistivity is decreased) as a result of the piezoresistive effect which is widely exploited^51^. As can be observed in Fig. 9, the dependence of the resistivity, used frequently as a quantity describing the performance of piezoresistive sensors, at various compression rates for both neat and filled matrices at different concentration and reprocessing cycles is presented. The resistivity is decreased at higher compression strains. For the neat TPU matrix, once a compression of 5% is achieved, a steep decrease of resistivity is apparent which is followed by a saturation as the samples underwent further deformation. Moreover, the matrices filled with the CI particles showed a notable increase in conductivity. The latter remark has been observed before in anisotropic MREs ^13^. In the same study it was suggested that the decrease of resistivity was achieved with a different mechanism known as the variable-range hopping mechanism.

Overall, piezoresistivity increases with concentration and compression, nevertheless it slightly decreases with recycling. A possible cause could be the created bonds between iron and matrix during reprocessing. Finally, the resistivity increases after each recycling process, however, the difference is not as significant resulting in an acceptable for an application.

## Conclusion

We present a new type of MREs, which are able to be reprocessed several times while keeping their mechanical properties in the same level. Together with their pure analogues these MREs were studied in terms of mechanical properties, internal structure, magnetorheological performance and piezoresistivity. As in any other polymer filled with stiff particles, the material becomes stiffer and stronger however, more ductile. Reprocessing the samples results in a very minor drop (less than 10%) of mechanical properties. In addition, the magnetic performance of the sample can be compared with similar TPU based MREs, however after recycling the MR effect is severely reduced. The structural changes of the material during reprocessing is the key to answer the drops in the magnetomechanical performance. On one hand, a supressed degradation still resulted in a minor chain shortening which was detected via GPC and resulted in the drop of stiffness. On the other hand, for the first time, the bonding between the particles and the matrix was shown with a convincing analysis during which the reprocessing procedure was simulated. Moreover, these specific MREs did not suffer from the Payne effect unlike a typical highly filled matrix. Finally, piezoresistivity tests confirmed that after each reprocessing cycle, the conductivity slightly fell, however that change does not significantly affect the final product. In practice, the amount of the recycled matrix does not exceed one third of the final product. On the contrary, in this work, the samples containing 100% of recycled TPUs were examined with no significant decrease in observed properties. It can be concluded that MREs based on TPU are suitable for reuse with the exception of their magnetorheological properties which can be boosted regardless using established procedures such as adding plasticizers or an oil.

## Data Availability

The datasets generated and/or analysed during the current study are not publicly available due to their use as a part of an ongoing study but are available from the corresponding author on reasonable request.

## References

[1] Ahamed R, Choi SB, Ferdaus MM (2018). A state of art on magneto-rheological materials and their potential applications. J. Intell. Mater. Syst. Struct..

[2] Saleh TA, Fadillah G, Ciptawati E (2021). Smart advanced responsive materials, synthesis methods and classifications: From lab to applications. J. Polym. Res..

[3] Carlson JD, Jolly MR (2000). MR fluid, foam and elastomer devices. Mechatronics.

[4] Sun S, Yang J, Du H, Zhang S, Yan T, Nakano M (2018). Development of magnetorheological elastomers-based tuned mass damper for building protection from seismic events. J. Intell. Mater. Syst. Struct..

[5] Komatsuzaki T, Inoue T, Terashima O (2016). Broadband vibration control of a structure by using a magnetorheological elastomer-based tuned dynamic absorber. Mechatronics.

[6] Cvek M, Moucka R, Sedlacik M, Babayan V, Pavlinek V (2017). Enhancement of radio-absorbing properties and thermal conductivity of polysiloxane-based magnetorheological elastomers by the alignment of filler particles. Smart Mater. Struct..

[7] Cvek M, Kutalkova E, Moucka R, Urbanek P, Sedlacik M (2020). Lightweight, transparent piezoresistive sensors conceptualized as anisotropic magnetorheological elastomers: A durability study. Int. J. Mech. Sci..

[8] Fiorillo AS, Critello CD, Pullano SA (2018). Theory, technology and applications of piezoresistive sensors: A review. Sens. Actuators A..

[9] Behrooz M, Gordaninejad F (2016). Three-dimensional study of a one-way, flexible magnetorheological elastomer-based micro fluid transport system. Smart Mater. Struct..

[10] Behrooz M, Gordaninejad F (2016). A flexible micro fluid transport system featuring magnetorheological elastomer. Smart Mater. Struct..

[11] Murao S, Mitsufuji K, Hirata K, Miyasaka F (2018). Coupled analysis by viscoelastic body with rigid body for design of MRE soft actuator. Electr. Eng. Jpn..

[12] Fu Y, Yao JJ, Zhao HH, Zhao G, Wan ZS, Guo RZ (2020). A muscle-like magnetorheological actuator based on bidisperse magnetic particles enhanced flexible alginate-gelatin sponges. Smart Mater. Struct..

[13] Kelley CR, Kauffman JL (2021). Towards wearable tremor suppression using dielectric elastomer stack actuators. Smart Mater. Struct..

[14] Stoll A, Mayer M, Monkman GJ, Shamonin M (2014). Evaluation of highly compliant magneto-active elastomers with colossal magnetorheological response. J. Appl. Polym. Sci..

[15] Plachy T, Kutalkova E, Sedlacik M, Vesel A, Masar M, Kuritka I (2018). Impact of corrosion process of carbonyl iron particles on magnetorheological behavior of their suspensions. J. Ind. Eng. Chem..

[16] Murin IV, Smirnov VM, Voronkov GP, Semenov VG, Povarov VG, Sinel'nikov BM (2000). Structural-chemical transformations of alpha-Fe_2_O_3_ upon transport reduction. Solid State Ionics.

[17] Cvek M, Mrlik M, Sevcik J, Sedlacik M (2018). Tailoring performance, damping, and surface properties of magnetorheological elastomers via particle-grafting technology. Polymers.

[18] Perez LD, Zuluaga MA, Kyu T, Mark JE, Lopez BL (2009). Preparation, characterization, and physical properties of multiwall carbon nanotube/elastomer composites. Polym. Eng. Sci..

[19] Boczkowska A, Awietjan SF, Wroblewski R (2007). Microstructure-property relationships of urethane magnetorheological elastomers. Smart Mater. Struct..

[20] Ju BX, Tang R, Zhang DY, Yang BL, Yu M, Liao CR (2016). Dynamic mechanical properties of magnetorheological elastomers based on polyurethane matrix. Polym. Compos..

[21] Grigorescu RM, Ghioca P, Iancu L, David ME, Andrei ER, Filipescu MI (2020). Development of thermoplastic composites based on recycled polypropylene and waste printed circuit boards. Waste Manage..

[22] Datta S, Naskar K, Bhardwaj YK, Sabharwal S (2011). A study on dynamic rheological characterisation of electron beam crosslinked high vinyl styrene butadiene styrene block copolymer. Polym. Bull..

[23] Wolfel B, Seefried A, Allen V, Kaschta J, Holmes C, Schubert DW (2020). Recycling and reprocessing of thermoplastic polyurethane materials towards nonwoven processing. Polymers.

[24] Vatandoost H, Rakheja S, Sedaghati R (2021). Effects of iron particles' volume fraction on compression mode properties of magnetorheological elastomers. J. Magn. Magn. Mater..

[25] Winger J, Schumann M, Kupka A, Odenbach S (2019). Influence of the particle size on the magnetorheological effect of magnetorheological elastomers. J. Magn. Magn. Mater..

[26] Kwon SH, An JS, Choi SY, Chung KH, Choi HJ (2019). Poly(glycidyl methacrylate) coated soft-magnetic carbonyl iron/silicone rubber composite elastomer and its magnetorheology. Macromol. Res..

[27] Kwon SH, Lee CJ, Choi HJ, Chung KH, Jung JH (2019). Viscoelastic and mechanical behaviors of magneto-rheological carbonyl iron/natural rubber composites with magnetic iron oxide nanoparticle. Smart Mater. Struct..

[28] Sorokin VV, Ecker E, Stepanov GV, Shamonin M, Monkman GJ, Kramarenko EY (2014). Experimental study of the magnetic field enhanced Payne effect in magnetorheological elastomers. Soft Matter.

[29] Fuensanta M, Martin-Martinez JM (2021). Structural and viscoelastic properties of thermoplastic polyurethanes containing mixed soft segments with potential application as pressure sensitive adhesives. Polymers.

[30] Albozahid M, Naji HZ, Alobad ZK, Saiani A (2021). TPU nanocomposites tailored by graphene nanoplatelets: The investigation of dispersion approaches and annealing treatment on thermal and mechanical properties. Polym. Bull..

[31] Luo Y, Xie YH, Geng W, Dai GF, Sheng XX, Xie DL (2022). Fabrication of thermoplastic polyurethane with functionalized MXene towards high mechanical strength, flame-retardant, and smoke suppression properties. J. Colloid Interface Sci..

[32] Bozyel I, Keser YI, Gokcen D (2021). Triple mode and multi-purpose flexible sensor fabrication based on carbon black and thermoplastic polyurethane composite with propolis. Sens. Actuators A..

[33] Wu JK, Gong XG, Chen L, Xia HS, Hu ZG (2009). Preparation and characterization of isotropic polyurethane magnetorheological elastomer through in situ polymerization. J. Appl. Polym. Sci..

[34] Wei B, Gong XL, Jiang WQ (2010). Influence of polyurethane properties on mechanical performances of magnetorheological elastomers. J. Appl. Polym. Sci..

[35] Cookson JW (2022). Theory of the Piezo-resistive effect. Phys. Rev..

[36] Li W, Jin X, Han X, Li YR, Wang WY, Lin T (2021). Synergy of porous structure and microstructure in piezoresistive material for high-performance and flexible pressure sensors. ACS Appl. Mater. Interfaces..

[37] Georgopoulou A, Michel S, Vanderborght B, Clemens F (2021). Piezoresistive sensor fiber composites based on silicone elastomers for the monitoring of the position of a robot arm. Sens. Actuators A..

[38] Zhang YH, Xia ZB, Huang H, Chen HQ (2009). A degradation study of waterborne polyurethane based on TDI. Polym. Testing.

[39] Hentschel T, Munstedt H (2001). Kinetics of the molar mass decrease in a polyurethane melt: A rheological study. Polymer.

[40] Cvek M, Kracalik M, Sedlacik M, Mrlik M, Sedlarik V (2019). Reprocessing of injection-molded magnetorheological elastomers based on TPE matrix. Composites B.

[41] Lopez-Pamies O (2010). An exact result for the macroscopic response of particle-reinforced neo-Hookean solids. J. Appl. Mech. Trans. ASME..

[42] Schrodner M, Pflug G (2018). Magnetomechanical properties of composites and fibers made from thermoplastic elastomers (TPE) and carbonyl iron powder (CIP). J. Magn. Magn. Mater..

[43] Takahara A, Coury AJ, Hergenrother RW, Cooper SL (1991). Effect of soft segment chemistry on the biostability of segmented polyurethanes. I. In vitro oxidation. J. Biomed. Mater. Res..

[44] Grigoryeva OP, Fainleb AM, Shumskii VF, Vilenskii VA, Kozak NV, Babkina NV (2009). The effect of multi-reprocessing on the structure and characteristics of thermoplastic elastomers based on recycled polymers. Polym. Sci. A.

[45] Cvek M, Mrlik M, Ilcikova M, Mosnacek J, Munster L, Pavlinek V (2017). Synthesis of silicone elastomers containing silyl-based polymer grafted carbonyl iron particles: An efficient way to improve magnetorheological, damping, and sensing performances. Macromolecules.

[46] Rabindranath, R. & Bose, H., editors. On the mobility of iron particles embedded in elastomeric silicone matrix. *13th International Conference on Electrorheological Fluids and Magnetorheological Suspensions (ERMR)* (2012).

[47] Sulkowski WW, Danch A, Moczynski M, Radon A, Sulkowska A, Borek J (2004). Thermogravimetric study of rubber waste-polyurethane composites. J. Therm. Anal. Calorim..

[48] Dwan'isa JPL, Mohanty AK, Misra M, Drzal LT, Kazemizadeh M (2004). Novel soy oil based polyurethane composites: Fabrication and dynamic mechanical properties evaluation. J. Mater. Sci..

[49] Barnes HA (2003). A review of the rheology of filled viscoelastic systems. Rheol. Rev..

[50] Rueda MM, Auscher MC, Fulchiron R, Perie T, Martin G, Sonntag P (2017). Rheology and applications of highly filled polymers: A review of current understanding. Prog. Polym. Sci..

[51] Chung DDL (2020). A critical review of piezoresistivity and its application in electrical-resistance-based strain sensing. J. Mater. Sci..

